# Surface Stoichiometry and Depth Profile of Ti_*x*_-Cu_*y*_N_*z*_ Thin Films Deposited by Magnetron Sputtering

**DOI:** 10.3390/ma14123191

**Published:** 2021-06-09

**Authors:** Arun Kumar Mukhopadhyay, Avishek Roy, Gourab Bhattacharjee, Sadhan Chandra Das, Abhijit Majumdar, Harm Wulff, Rainer Hippler

**Affiliations:** 1 Department of Physics, Indian Institute of Engineering Science and Technology, Shibpur, Howrah 711103, India; akmphy_dac@rediffmail.com (A.K.M.); aviroy22@gmail.com (A.R.); majuabhijit@gmail.com (A.M.); 2 Department of Physics, Dinabandhu Andrews College, 54 Raja S.C. Mallick Road, Kolkata 700008, India; 3 Department of Electronics, Vidyasagar College, 39 Sankar Ghosh Lane, Kolkata 700006, India; 4 Surface Physics and Material Science Division, Saha Institute of Nuclear Physics, HBNI, 1/AF Bidhannagar, Kolkata 700064, India; gourab205@gmail.com; 5 UGC-DAE Consortium for Scientific Research, Indore 452017, India; scdascsr@gmail.com; 6 Institute of Physics, University of Greifswald, Felix-Hausdorff-Str. 6, 17489 Greifswald, Germany; wulff@uni-greifswald.de; 7 Institute of Physics, Czech Academy of Sciences, Na Slovance 2, 18221 Prague, Czech Republic

**Keywords:** magnetron sputtering, Ti-Cu-N coating, N incorporation, X-ray photoelectron spectroscopy, X-ray diffraction, Transmission electron microscopy

## Abstract

We report the surface stoichiometry of Ti${}_{x}$-Cu${}_{y}$N${}_{z}$ thin film as a function of film depth. Films are deposited by high power impulse (HiPIMS) and DC magnetron sputtering (DCMS). The composition of Ti, Cu, and N in the deposited film is investigated by X-ray photoelectron spectroscopy (XPS). At a larger depth, the relative composition of Cu and Ti in the film is increased compared to the surface. The amount of adventitious carbon which is present on the film surface strongly decreases with film depth. Deposited films also contain a significant amount of oxygen whose origin is not fully clear. Grazing incidence X-ray diffraction (GIXD) shows a Cu${}_{3}$N phase on the surface, while transmission electron microscopy (TEM) indicates a polycrystalline structure and the presence of a Ti${}_{3}$CuN phase.

## 1. Introduction

During the past decades, thermodynamically stable metal nitrides (MN) have been studied extensively for their electrical, magnetic, tribological, corrosion, and anti-bacterial properties and the resulting potential applications [1,2,3,4,5,6]. A major concern of these studies are the physical properties of MN by changing the atomic nitrogen percentage. However, in the last few years, the research on metastable nitrides, such as copper nitrides, has attracted much attention due to their decomposition above a certain temperature, which has been used in the fabrication of microscopic metal lines without involving a lithographic process [7,8,9]. Well-known phases of the Cu-N compound (not considering the unstable copper azide) are Cu${}_{3}$N and Cu${}_{4}$N, which exhibit a semiconductor-like and metallic behaviour, respectively [10,11]. Binary Ti-Cu compounds are well known for their antibacterial activity [4,5]. Ternary Ti-Cu-N compounds are rare on Earth but have been observed as crystalline CuTiN${}_{2}$ and Ti${}_{3}$CuN phases in meteorites [12].

Different methods to deposit metal nitride films have been employed, e.g., ion-assisted deposition [7], ion implantation [13], reactive pulsed laser deposition [14], and reactive radio-frequency (RF) magnetron sputtering [15,16]. Magnetron sputtering is a versatile technique for deposition of thin solid films. Low power direct current (DCMS) and high power impulse magnetron sputtering (HiPIMS) are frequently employed. HiPIMS results in much larger plasma densities, usually exceeding 10${}^{18}$ m${}^{-3}$, which is 2–3 orders of magnitude larger compared to DCMS [17,18]. In addition, plasma ions produced during HiPIMS are more energetic compared to conventional DCMS [19]. Major obstacles for the synthesis of Ti${}_{x}$-Cu${}_{y}$N${}_{z}$ films are the incorporation of nitrogen into the TiCu layer to reach an approximately equal Ti:Cu:N ratio and the formation of Ti-N and Cu-N bonds. Copper nitride has been widely studied but little information is available in the literature concerning Ti-doped Cu${}_{3}$N films, their surface stoichiometry and the proper concentration of individual elements [20].

The present work constitutes a feasibility study for deposition of TiCuN films. New results for TiCuN film deposition by magnetron sputtering are reported. Deposited films are investigated by means of X-ray photoelectron spectroscopy (XPS). The composition and chemical bond structure are investigated as a function of film depth. Film morphology and crystallinity are investigated by X-ray diffraction (XRD) and transmission electron microcopy (TEM).

## 2. Materials And Methods

### 2.1. Ti${}_{x}$-Cu${}_{y}$N${}_{z}$ Film Synthesis by Combination of DCMS and Hipims

**Figure 1:**
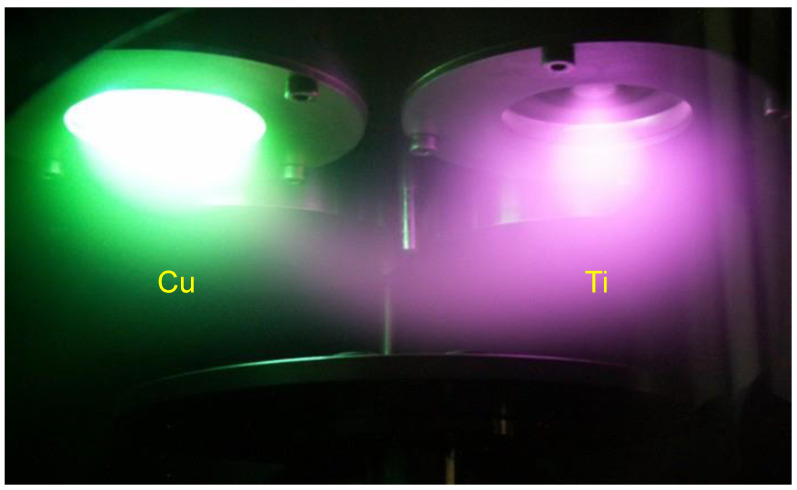
Photo showing both magnetrons with Cu and Ti targets during a HiPIMS pulse applied to the Cu cathode.

Ti${}_{x}$-Cu${}_{y}$N${}_{z}$ films are deposited by co-sputtering in a direct current/high power impulse magnetron sputtering (DCMS/HiPIMS) system using nitrogen (N${}_{2}$) as the working gas [20,21]. Two commercial planar magnetrons (SW 50, Gencoa, Liverpool, UK) with opposite magnetic field configurations were attached to a high vacuum chamber. The vacuum chamber was evacuated with the help of a turbomolecular pump to a base pressure greater than 1 ×$10^{-4}$ Pa. Cu and Ti targets with a 50 mm diameter and 6 mm thickness were sputtered at partial pressures of 1 Pa and 5 Pa at a N${}_{2}$ gas flow rate of 10 sccm. Each magnetron was separately powered by a power supply (Pinnacle 3000, Advanced Energy). The Cu target was sputtered in direct current magneton sputtering (DCMS) mode with a discharge current of 0.3 A and a discharge power P=100 W. The Ti target was sputtered in HiPIMS mode with a pulse length of 100 μs and a repetition frequency of 100 Hz. A home-build electronic power switch combined with a direct current power supply was employed [22,23,24]. The initial discharge voltage applied to the cathode (target) was set to –800 V, which resulted in a maximum discharge current of 35 A. It gave rise to a large fraction of sputtered Ti ions due to the high plasma density [17,18]. Figure 1 shows both magnetrons with a negative HiPIMS pulse applied to the Cu cathode. The HiPIMS plasma emerges from the Cu cathode and follows the magnetic field lines to the Ti cathode.

Ti${}_{x}$-Cu${}_{y}$N${}_{z}$ films were deposited on p–type Si(100) substrate. The deposition time was 1 h and the deposited film thickness was approximately 5 μm.

### 2.2. Ar Etching and Thickness Measurement

Deposited films were transported to a second chamber (ex-situ) where films were subjected to argon ion etching with an in-built etching gun (SPECS, PU-IQE 12/38, SPECS Surface Nano Analysis GmbH, Berlin, Germany) using argon (purity 99.99%) as working gas. Etching was performed at 2 keV with an emission current of 5 mA and ion current of 10 μA. The gas pressure during etching was about $2\times 10^{-6}$ mbar. The samples were etched for different time durations (11 min, 33 min, and 44 min). The etch rate was monitored with the help of a quartz crystal microbalance (QCM). Eroded material deposits on the QCM and the deposition rate were measured. A relation between deposition rate measured by the QCM and etch rate was established in a separate experiment. The measured etching rate was 90 nm/min.

### 2.3. Film Characterization

Deposited films were characterized by X-ray photoelectron spectroscopy (XPS). XPS measurements of Ti${}_{x}$-Cu${}_{y}$N${}_{z}$ films were performed with a multi–technique 100 mm hemispherical electron analyser (ESCA, VSW Atomtech Ltd., Witney, Oxfordshire, UK) using Al Kα radiation (photon energy 1486.6 eV) with a line width of 0.85 eV as the excitation source. XPS spectra were collected in constant analyser energy mode with a pass energy of 23.5 eV at 0.125 eV/step and a chamber pressure of 10${}^{-9}$ mbar [25]. The energy scale was calibrated using the adventitious C-1s peak with a binding energy of 284.5 eV. A combination of Lorentzian and Gaussian line shapes and a Shirley background were employed in the analysis.

Grazing incidence X-ray diffraction (GIXD) measurements were carried out to determine the phase composition of deposited films [26]. All measurements were performed with a θ–θ diffractometer (D 5000, Bruker AXS GmbH, Karlsruhe, Germany) using Cu-Kα radiation (λ=0.154 nm). The scanned 2θ range was 20–85${}^{\circ }$ at a constant incidence angle $\omega =1.0^{\circ }$. At these conditions, the vertical penetration depth of Cu-Kα radiation in pure copper, calculated with the help of X-ray mass attenuation coefficients, was approximately 400 nm [26,27].

The Ti${}_{x}$-Cu${}_{y}$N${}_{z}$ film deposited on p-type Si(100) substrate was prepared for the cross-section Transmission Electron Microscopy (TEM) analysis. For that, the sample was cut into two pieces which were bonded face to face using epoxy glue. The bonded sample was heated to approximately 400 K for 10 min under ambient conditions and put inside a brass tube with a diameter of 3 mm. Empty spaces inside the tube were filled with dummy Si wafer and glue. The tube was cut into small discs with a thickness of 0.5 mm using a diamond wire cutter. The sample was thinned to approximately 110 nm and polished by mechanical thinning followed by double dimpling and final milling by Ar${}^{+}$ ions using a precision ion polishing system (PIPS, GATAN inc, Pleasanton, CA, USA) with an Ar${}^{+}$ ion energy of 3 keV. TEM studies were carried out using a TEM (FEI Tecnai G2 F30-ST, Hillsboro, OR, USA) operated at 300 keV and equipped with a high-angle annular dark field (HAADF) detector (Fischione, model 3000) and an energy dispersive X-ray spectroscopy (EDX) attachment for compositional analysis. For TEM observation, the samples were aligned along.

## 3. Results And Discussion

### 3.1. Chemical Composition and Bond Structure

**Figure 2:**
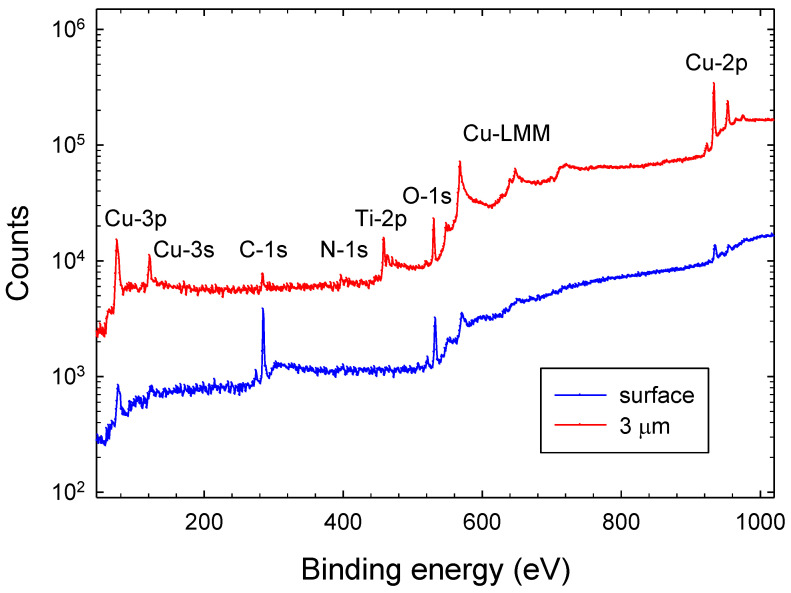
Full-scale (40 eV to 1020 eV) XPS spectrum of a deposited Ti${}_{x}$-Cu${}_{y}$N${}_{z}$ thin film of the as-deposited film (surface) and at a film depth of 3 μm.

The chemical composition and bond structure of the Ti-CuN film is derived from XPS measurements. Figure 2 shows two XPS survey scans (40 eV to 1020 eV) of a Ti${}_{x}$-Cu${}_{y}$N${}_{z}$ film at different depths from the film surface. The lower plot shows results of the as-deposited film, while the upper plot is from a film depth of 3 μm. Observed photoelectron lines orginate from C-1s, N-1s, O-1s, Ti-2p, and Cu-2p core levels. Lines from Cu-3p and Cu-3s as well as prominent Cu LMM Auger lines at 568 eV (L${}_{3}$M${}_{45}$M${}_{45}$), 648 eV (L${}_{3}$M${}_{23}$M${}_{45}$), and 720 eV (L${}_{3}$M${}_{23}$M${}_{23}$) are observed. It is immediately noted that the as-deposited (surface) sample contains a significant amount of (adventitious) carbon (C) on its surface, where metal (Cu, Ti) and nitrogen (N) contributions are rather small. Adventitious carbon is a well-known phenomenon in XPS as carbonaceous layers already form during short exposures to an ambient atmosphere [28]. Strong XPS metal lines from Cu and Ti appear after etching (3 μm). In addition, in all depths, a significant amount of oxygen is noted. Some oxygen contamination may have occurred during the deposition process. We believe, however, that oxidation of the deposited film during storage and ex-situ transport from the deposition to the analysis chamber (which took several weeks) is the main cause for the oxygen impurity. Oxidation of metal nitride films is a well-known phenomenon and several investigations of the oxidation behaviour have been reported recently [29,30,31].

**Figure 3:**
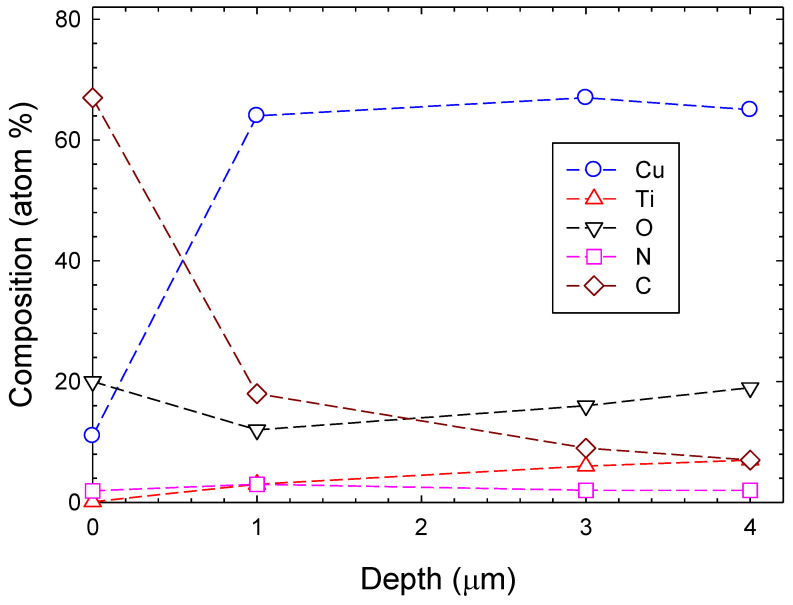
Relative composition (in atom %) as a function of film depth of the deposited Ti-CuN film. Dashed lines are to guide the eye only.

The relative composition of the deposited film as a function of depth as derived from XPS individual peak scans making use of integrated peak intensities after subtraction of a Shirley background and taking atomic sensitivity factors into account is displayed in Figure 3. It is noted that the film surface is contaminated with (adventitious) carbon. As a consequence, little Cu and Ti is found at the surface. The amount of copper strongly increases with film depth, reaching approximately 65% at depths of 1–4 μm. The contribution of Ti is rather small right at the surface; it amounts to about 7% at the largest depth. It is obvious that the Cu and Ti compositions of the films are very different. The large difference is explained by a different sputtering yield, which is about four times larger for Cu compared to Ti. The nitrogen composition amounts to approximately 2% throughout the film. Thge oxygen contribution is rather large and amounts to approximately 20%.

**Figure 4:**
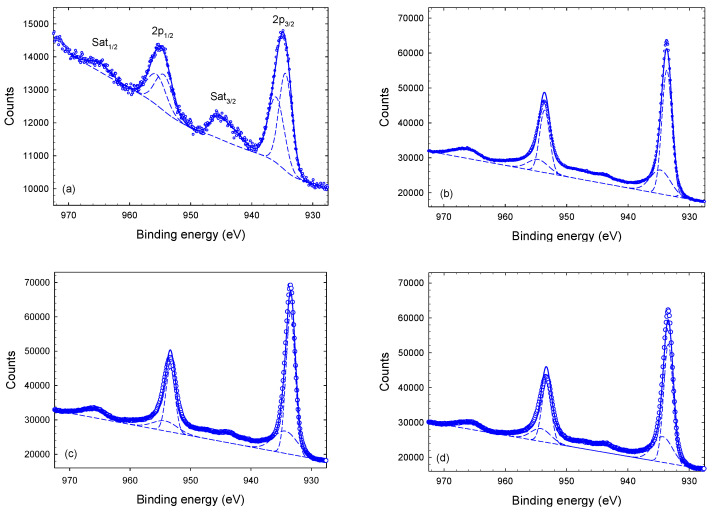
XPS spectra of Cu-2p core peak of Ti${}_{x}$-Cu${}_{y}$N${}_{z}$ film at different depths; where (**a**) at surface, (**b**) 1 μm, (**c**) 3 μm and (**d**) 4 μm.

The deconvolution of the individual lines provides information related to possible chemical bonds of the investigated elements. Figure 4 shows Cu-2p XPS spectra of the as-deposited (surface) coating and of the etched sample taken at depths of 1 μm, 3 μm, and 4 μm. Cu-2p shows two main peaks near 934 eV and 954 eV separated by 19.9 eV which originate from the Cu-2p${}_{3/2}$ and Cu-2p${}_{1/2}$ sub-shells, respectively. An intensity ratio of 2:1 is assumed for the Cu-2p${}_{3/2}$ and Cu-2p${}_{1/2}$ sub-peaks. In addition, shake-up satellite peaks (Sat${}_{1/2}$ and Sat${}_{3/2}$), as indicated in Figure 4a, are present in all of the Cu-2p XPS spectra. Figure 4a was fitted with a Shirley background, yielding a reasonable fit. As is obvious from Figure 4b–d, the same procedure did not work as well for the etched samples. In these fits, we used a linear background as a Shirley background did not provide reasonable results. Nevertheless, the quality of the fits is still insufficent, which we partly attribute to the incorrect background under the satellite peaks. Satellite peaks are relatively small for the etched samples compared to the surface sample. It resembles the fact that satellite peaks originate from CuO states which dominate the surface XPS spectrum.

**Figure 5:**
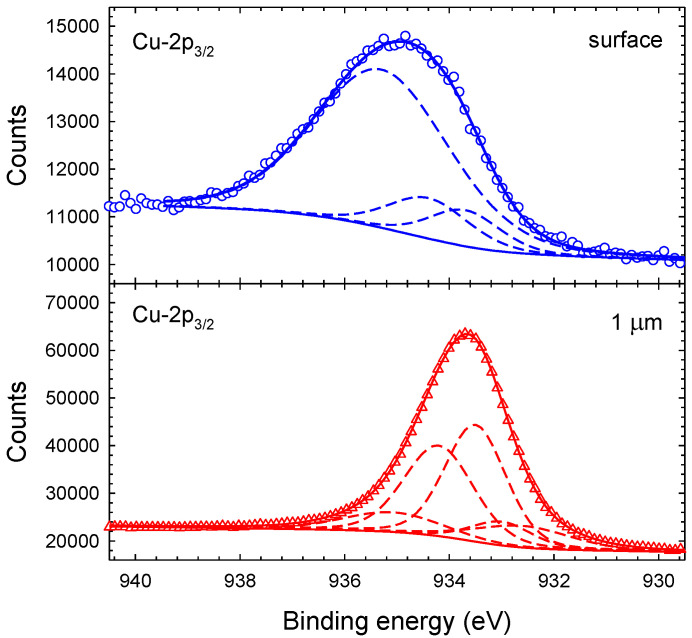
Cu-2p${}_{3/2}$ peak fitted with 5 sub-peaks (see text) at the surface (**top**) and at a film depth of 1 μm (**bottom**). Data are fitted with five Lorentzian–Gaussian sub-peaks after subtraction of a Shirley background.

According to Biesinger et al., Cu-2p lines from metallic Cu(0) and Cu(I) oxide appear at 932.6 eV and 932.2 eV, respectively, while Cu(II) oxide and hydroxide contribute at 933.8 eV and 934.7 eV, respectively [32,33]. Cu-N bonds are expected to contribute at 933.1 eV and with the same line shape [34]. Each of the main Cu-2p${}_{3/2}$ and Cu-2p${}_{1/2}$ peaks, hence, should be fitted with (at least) five sub-peaks. The results of such fits with five Lorentzian–Gaussian sub-peaks each are shown in Figure 5 for the Cu-2p${}_{3/2}$ peak of the as-deposited film (surface) and at a depth of 1 μm after etching. Evidently, the etched (depth 1 μm) film is well represented by five sub-peaks; the relative intensities and the full-width-at-half-maximum (FWHM) obtained with this fit are given in Table 1. Similar fits for the Cu-2p${}_{3/2}$ main peak are obtained for the etched sample at depths of 3 μm and 4 μm. The surface XPS spectrum is shifted by approximately 1.3 eV to larger binding energy values compared to the etched sample. Fixing the Cu(0) peak position at 933.1 eV (±0.1 eV), which is the average value of the etched samples, we obtain the fit shown in Figure 5 for the as-deposited coating. We mention in passing that the extracted peak position is, hence, 0.5 eV larger compared to Biesinger et al. [32]. We want to emphasize that all spectra were fitted with five sub-peaks even though the surface spectrum is represented by three sub-peaks only (two of the five sub-peaks were returned with zero intensity by the fit). The results show that the surface is composed of Cu(II) oxide and hydroxide (Table 1). This interpretation is further confirmed by the Cu shake-up satellite lines, which are attributed to copper oxide Cu(II)O and Cu(II)OH [32]. The width of the Cu(II) hydroxide peak is significantly larger compared to other peaks, which agrees with the results of Biesinger et al. [32].

**Figure 6:**
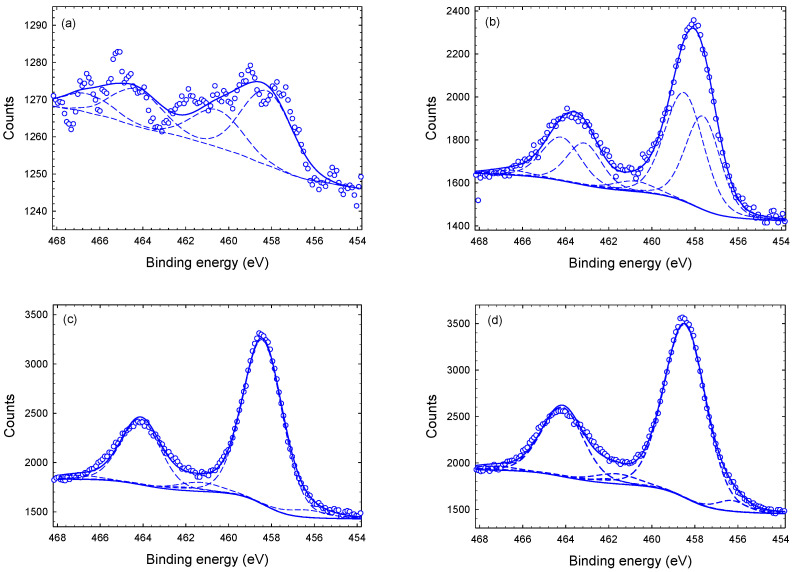
Typical Ti-2p XPS spectra of Ti${}_{x}$-Cu${}_{y}$N${}_{z}$ film at different depths: (**a**) at the surface (smoothed), (**b**) 1 μm, (**c**) 3 μm, and (**d**) 4 μm. The data are fitted with Lorentzian–Gaussian peaks after subtraction of a Shirley background.

XPS spectra of Ti-2p are displayed in Figure 6. Ti-2p shows two main peaks related to the Ti-2p${}_{1/2}$ and Ti-2p${}_{3/2}$, which are separated by 5.7–6.1 eV depending on the chemical state [32]. From the deconvolution of each main peak into several sub-peaks, we find for all four samples an almost identical position of the main sub-peak at approximately 458.6 eV corresponding to Ti(IV) oxide (Table 2). A second sub-peak at approximately 457 eV is related to Ti(III) oxide [32]. Ti-N and Ti(II)-O bonds contributing at 455 and 455.3 eV, respectively, are not observed [32,35]. The origin of the third sub-peak at approximately 461.5 eV is not yet understood. It is well known that the high reactivity of TiN with oxygen can lead to the formation of oxy-nitride overlayers [36,37]. As this peak is rather small, approximately 5%, it might be an artefact caused by some asymmetry of the other two sub-peaks.

**Figure 7:**
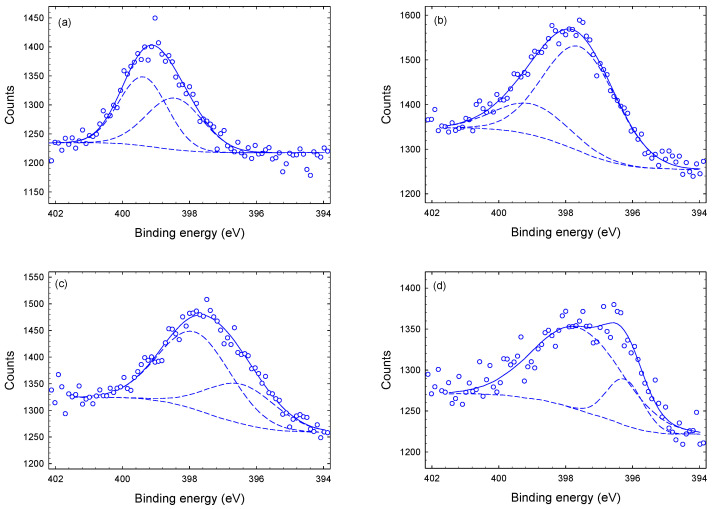
Deconvoluted N-1s XPS spectra of Ti${}_{x}$-Cu${}_{y}$N${}_{z}$ film at different depths: (**a**) at the surface, (**b**) 1 μm (**c**) 3 μm and (**d**) 4 μm.

Figure 7 displays the deconvoluted N-1s XPS spectra. All N-1s spectra display a pronounced peak at approximately 398 eV which is associated with Cu-N bonds [20,38]. At the larger depths (1–4 μm), a pronounced peak at approxcimately 397 eV appears, which is assigned to Ti-N bonds. The main sub-peak of the surface spectrum at approximately 399.5 eV is presumably due to oxy-nitrides (N-O) and/or C-N bonds [35,39,40].

**Figure 8:**
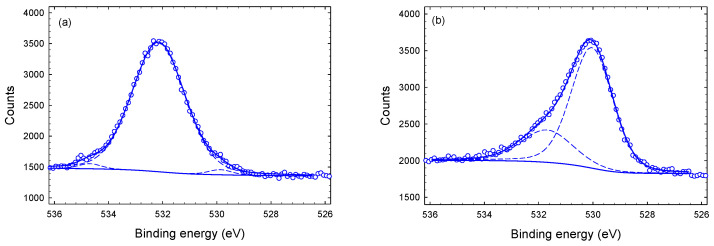

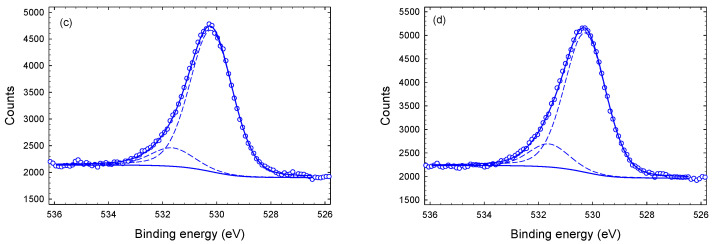
O-1s core level XPS spectra of Ti${}_{x}$-Cu${}_{y}$N${}_{z}$ film at different depth: (**a**) at the surface, (**b**) 1 μm (**c**) 3 μm and (**d**) 4 μm.

Figure 8 shows deconvoluted O-1s spectra. O-1s spectra of the etched samples (1–4 μm) show a main sub-peak at 530.1 eV and a smaller sub-peak at approximately 531.7 eV, both with a typical width of approximately 2.0 eV. The positions are independent of the depth at which the spectra were taken. The two sub-peaks at 530.1 eV and 531.7 eV are attributed to metal (Cu or Ti) oxide and Cu hydroxide, respectively [32]. The surface O-1s spectrum is shifted by approximately 2.0 eV compared to the etched sample. It is composed of a main sub-peak at 532.1 eV and two small side peaks at 529.9 eV and 534.5 eV (Figure 8a). The small sub-peak at 529.9 eV is again associated with metal oxide. The main sub-peak at 532.1 eV with a width of 2.3 eV is somewhat broader than expected. It appears likely that in reality this sub-peak is composed of two sub-peaks which are comparable in size and separated by about 1 eV from each other. In light of this, we can relate the sub-peak to metal hydroxide (e.g., Cu-OH) as well as to C-O bonds with adventitious carbon. The remaining small sub-peak at 534 eV could be caused by water contamination at the surface [41].

### 3.2. GIXD of Ti${}_{x}$-Cu${}_{y}$N${}_{z}$ Film

**Figure 9:**
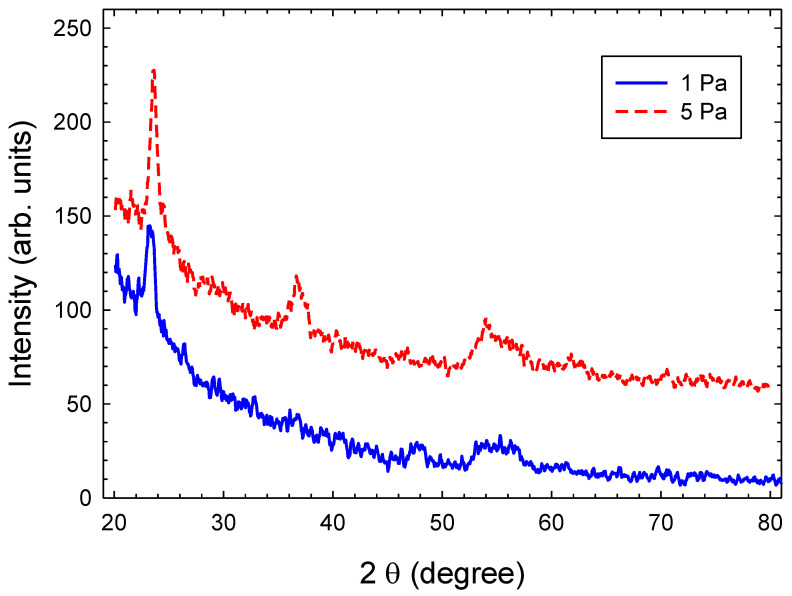
GIXD pattern of Ti${}_{x}$-Cu${}_{y}$N${}_{z}$ films deposited at 1 Pa and 5 Pa.

Grazing incidence X-ray diffraction (GIXD) measurements of Ti${}_{x}$-Cu${}_{y}$N${}_{z}$ films deposited at gas pressures of 1 and 5 Pa are displayed in Figure 9. These measurements, hence, are sensitive to the crystallinity of the surface layers. The figure shows a diffraction pattern superimposed onto a large background. However, the observed X-ray pattern cannot provide unambiguous information on the chemical composition of crystalline phases. The peak-to-background ratio is small and and the amorphous amount as inferred from the large background is substantial. The observed peaks are strongly broadened and several line overlaps are observable. The X-ray pattern of the film deposited at 1 Pa shows a few reflections connected to Cu${}_{3}$N [20] but with a strongly preferred [h00] orientation and two small reflections which are not clearly allocated to chemical compounds according to the XPS results. The qualitative X-ray phase analysis of the 5 Pa film also yields no clear result. At least three reflections should match with any ICDD (ICDD-PDF2) reference phase. In accordance with XPS results, it appears most likely that Cu(OH)${}_{2}$ and copper oxides are the dominant phases at the surface [20,42,43]. The origin of the broad reflection peak at 54–57${}^{\circ }$ is not that clear. It may be related to TiO${}_{2}$ (rutile, anatase, or brookite), which has several diffraction peaks in this range [44]. Reflections from a rutile TiO${}_{2}$ phase should occur at peak positions of 54.3${}^{\circ }$ (211) and 56.6${}^{\circ }$ (220), from anatase TiO${}_{2}$ at 53.9${}^{\circ }$ and 55.1${}^{\circ }$ (211), and from brookite TiO${}_{2}$ at 54.2${}^{\circ }$ (023), 55.2${}^{\circ }$ (142) and 57.1${}^{\circ }$ (311). However, these peaks are usually not the most intensive. The diffraction pattern of a small crystallite ensemble depends on the scattering behaviour of the irradiated grains and requires an orientation averaging. In XRD investigations of thin films obtained in technological processes, the orientation averaging of the crystallite scattering is significantly influenced by the microscopical structure of the sample material and it depends, in a complicated manner, on the spatial phase distribution and on the substructure, e.g., grain size, grain orientation, and internal stress. Reflections from Ti${}_{x}$Cu${}_{y}$ metallic phases appear unlikely. The main reflection pattern of Ti${}_{x}$Cu${}_{y}$ is expected in the angular range 39–45${}^{\circ }$ but are not observed [45].

**Figure 10:**
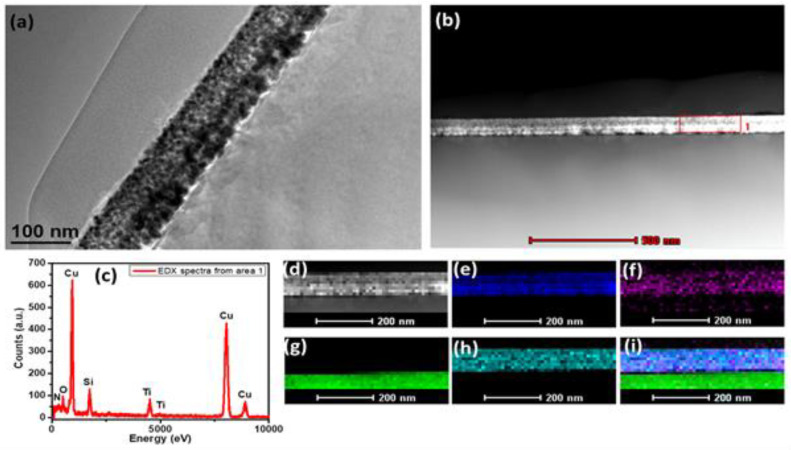
(**a**) Bright field TEM image, (**b**) STEM HAADF image, (**c**) EDX spectrum, (**d**) STEM image of mapping area, (**e**) Cu-K map, (**f**) N-K map, (**g**) Si-K map, (**h**) Ti-K map, (**i**) composite map of Ti${}_{x}$-Cu${}_{y}$N${}_{z}$ film.

A bright field TEM image of the Ti${}_{x}$-Cu${}_{y}$N${}_{z}$ film is displayed in Figure 10a. Unlike GIXD, the present TEM results represent deeper layers of the deposited film. The investigated sample was prepared by mechanical thinning followed by double dimpling and Ar${}^{+}$ ion milling, as explained in Section 2; the thickness of the sample after thinning is approximately 110 nm. A STEM HAADF image of the film is shown in Figure 10b and the corresponding EDX spectrum, which is collected from the indicated area in the insert of that figure, is shown in Figure 10c. The spectrum shows that the elements Cu, Ti, O, and N are present in the sample. The Si signal is from the Si substrate. The extracted Cu/Ti ratio is 11.7, which agrees well with a Cu/Ti ratio of 11.2 obtained from the XPS measurements for a film depth of 3 μm (Figure 3). The corresponding chemical mapping was performed for the selected area shown in Figure 10d. The acquired Cu-K, Ti-K, N-K, Si-K, and the composite map are shown in Figure 10e–i, respectively. From the chemical mapping, we can clearly see that the film contains Cu, Ti, O, and little N on a substrate composed of Si.

### 3.3. TEM and EDX of Ti${}_{x}$-Cu${}_{y}$N${}_{z}$ Film

**Figure 11:**
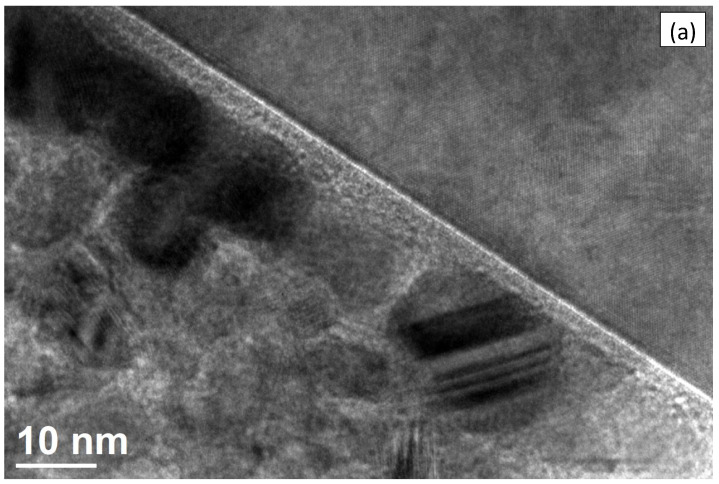

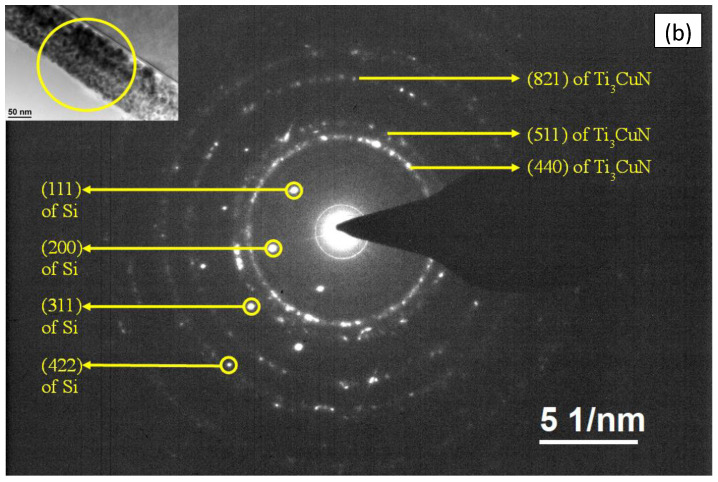
(**a**) HRTEM image, (**b**) SAED pattern of Ti${}_{x}$-Cu${}_{y}$N${}_{z}$ film. Insert shows investigated region.

The HRTEM image of the Ti${}_{x}$-Cu${}_{y}$N${}_{z}$ film on the Si substrate is shown in Figure 11a. From the HRTEM image, we see that the Ti${}_{x}$-Cu${}_{y}$N${}_{z}$ film is composed of small grains with a typical size of about 10–15 nm. It partly explains, as is evident from Figure 10f,h, why the elemental composition of N and Ti is not uniform. The contribution of these elements to the film composition is larger inside the grains and lower outside.

The interplanar spacings of different planes present in our sample are obtained from a selected area electron diffraction (SAED) pattern. If the distance of any spot from the central bright spot or the radius of any circle is $R_{hkl}$, then the interplanar distance is given by $d_{hkl}=\lambda \;L/R_{hkl}$, where [hkl] are the Miller indices of the particular plane, λ is the calculated electron wavelength, and *L* is the effective camera length. The measured interplanar distances for three circles are 0.2118 nm, 0.1969 nm, and 0.1296 nm, which almost match with the interplanar spacings of [440], [610], and [821], respectively, planes of Ti${}_{3}$CuN (JCPDS No 50-1475), which is polycrystalline in nature. The measured interplanar distances of the dots are 0.3173 nm and 0.2714 nm, which almost match with the interplanar spacings of [111] and [200] planes, respectively, of the Si (JCPDS No 80-0018) substate, which is a single crystal.

The TEM result of a Ti${}_{3}$CuN crystallographic phase is distinctly different from the GIXD result where the observed pattern is attributed to Cu${}_{3}$N phase. There are a few stable stable phases belonging to the Ti-Cu-N ternary system, in particular Cu${}_{3}$N, Ti${}_{3}$CuN, and CuTiN${}_{2}$ [12]. The CuTiN${}_{2}$ phase appears unlikely as it requires a large N content, which is not present in the films at hand. One may speculate that the difference between GIXD and TEM results is presumably caused by a different film treatment. While the GIXD sample is an as-deposited film, the TEM sample has been treated by mechanical thinning and by a mild heat treatment—both in ambient air—which, in combination, may have caused a transition from a Cu${}_{3}$N to a Ti${}_{3}$CuN phase.

## 4. Conclusions

Ti${}_{x}$Cu${}_{y}$N${}_{z}$ thin films were deposited by magnetron co-sputtering at gas pressures of 1 and 5 Pa in a nitrogen atmosphere. The deposited films are composed of Cu, Ti, and N and contain a large O contamination as well as adventitious C on the film surface. The composition was investigated as a function of film depths. Cu has the largest contribution of more than 60% at film depths of 1–3 μm. XPS measurements indicate that the top layers contain the Cu(OH)${}_{2}$ phase as well as other phases, which are attributed to oxidized CuO and/or Cu${}_{2}$O. Composition of Ti and N is comparatively small, about 5% and 2%, respectively. The small Ti content is explained by the considerably smaller sputtering yield of Ti compared to Cu. The low nitrogen content indicates that formation of nitrogen-containing compounds is less favourable under the given conditions. In addition, as indicated by the large surface oxygen fraction, a significant post-deposition oxidation during storage of the films in ambient air may have occurred.

Film morphology was investigated by GIXD and TEM. GIXD is more sensitive to the top surface layers, which contain the Cu${}_{3}$N phase. Deeper layers, as investigated by TEM after mechanical thinning and heating to about 400 K, indicate the presence of a polycrystalline Ti${}_{3}$CuN phase.

It is our intention to continue the present research, in particular, using different deposition conditions to change the composition and with respect to the oxidation behaviour of deposited films.

## Figures and Tables

**Table 1:** Line positions relative to Cu(0) peak, FWHM (full-width-at-half-maximum), and relative peak intensity of Cu-2p${}_{3/2}$ XPS sub-peaks of the deposited Ti${}_{x}$-Cu${}_{y}$N${}_{z}$ coating for different film depth.

	Position (eV)	FWHM (eV)				Relative Intensity (%)			
		Surface	1 μm	3 μm	4 μm	Surface	1 μm	3 μm	4 μm
Cu(0)	933.1 (±0.1)	–	1.4	1.3	1.3	–	8	21	27
Cu(I)O	−0.4	–	2.1	2.0	2.1	–	11	15	19
Cu(II)O	+1.2	2.0	1.6	1.3	1.5	16	33	24	24
CuN	+0.5	1.4	1.4	1.3	1.3	10	38	33	24
Cu-OH	+2.0	3.1	2.2	2.6	2.6	74	10	7	6

**Table 2:** Position, FWHM (full-width-at-half-maximum), and relative peak intensity of Ti-2p${}_{3/2}$ XPS sub-peaks of the deposited Ti${}_{x}$-Cu${}_{y}$N${}_{z}$ coating for different film depth.

	Position (eV)	FWHM (eV)				Relative Intensity (%)			
		Surface	1 μm	3 μm	4 μm	Surface	1 μm	3 μm	4 μm
Sub-Peak I	456.9 (±0.6)	2.3	1.9	2.3	1.6	35	41	4	4
Sub-Peak II	458.6 (±0.2)	2.3	2.1	2.2	2.3	62	54	91	91
Sub-Peak III	461.6 (±0.4)	1.3	2.2	2.4	2.4	4	5	5	5

## Data Availability

The data presented in this study are available on request from the corresponding author.

## Abbreviations

The following abbreviations are used in this manuscript:

DCMS	Direct current magnetron sputtering
EDX	Energy dispersive X-ray spectroscopy
GIXD	Grazing incidence X-ray diffraction
HAADF	High-angle annular dark field
HiPIMS	High power impulse magnetron sputtering
HRTEM	High resolution TEM
SAED	Selected area electron diffraction
TEM	Transmission electron microscopy
XPS	X-ray photoelectron spectroscopy
XRD	X-ray diffraction
